# Association between hemoglobin glycation index and metabolic syndrome in Saudi adolescents: A cross-sectional study

**DOI:** 10.1097/MD.0000000000051375

**Published:** 2026-11-06

**Authors:** Sobhy M. Yakout, Malak N.K. Khattak, Abdullah M Alnaami, Nasser M. Al-Daghri

**Affiliations:** a Department of Biochemistry, Chair for Biomarkers of Chronic Diseases, College of Science, King Saud University, Riyadh, Saudi Arabia.

**Keywords:** adolescents, cardiometabolic risk, fasting plasma glucose, glycated Hemoglobin, Hemoglobin glycation index, Metabolic syndrome, obesity

## Abstract

Metabolic syndrome (MetS) is a cluster of cardiometabolic risk factors associated with an increased risk of cardiovascular disease and type 2 diabetes mellitus. The Hemoglobin Glycation Index (HGI), which quantifies the difference between observed glycated hemoglobin (HbA1c) and HbA1c predicted from fasting plasma glucose, has been proposed as a metabolic risk marker. However, evidence regarding its association with MetS remains inconsistent, particularly among adolescents. This study examined the associations between HGI and MetS, including sex-stratified patterns, among Saudi adolescents from Riyadh. This cross-sectional study included 2342 Saudi adolescents (893 boys and 1449 girls; mean age, 14.8 ± 1.7 years) recruited from secondary schools in Riyadh between September 2019 and March 2021. Anthropometric measurements, blood pressure, fasting plasma glucose, HbA1c, lipid profile, vitamin D, and selected biochemical markers were assessed. HGI was calculated as the difference between observed and predicted HbA1c, with predicted values derived from a linear regression model using fasting plasma glucose. MetS was defined using established pediatric criteria. Unadjusted binary logistic regression and correlation analyses were performed. MetS was present in 43.3% of participants and was more prevalent in boys than girls (47.1% vs 40.9%). Participants with MetS had higher body mass index, blood pressure, triglycerides, and fasting plasma glucose levels, lower high-density lipoprotein cholesterol and vitamin D levels (all *P* <.01). HGI was lower in participants with MetS than those without (−0.057 ± 0.60 vs −0.003 ± 0.52, *P* <.05), with the largest difference observed among girls. In unadjusted logistic regression analyses, higher HGI tertiles were associated with lower odds of MetS compared with the lowest tertile. Correlation analyses revealed different correlation patterns between boys and girls; however, most associations between HGI and clinical or biochemical variables were weak in magnitude. Higher HGI tertiles were inversely associated with MetS in this cross-sectional sample of Saudi adolescents, contrary to patterns commonly reported in adult populations. As these associations were unadjusted and the study was cross-sectional, they should not be interpreted as independent or causal. Longitudinal studies incorporating direct measures of insulin resistance, pubertal status, erythrocyte turnover, and inflammatory markers are needed to clarify the biological basis and directionality of the HGI–MetS association in adolescents.

## 1. Introduction

Metabolic syndrome (MetS) is a cluster of cardiometabolic risk factors (central obesity, dyslipidaemia, hypertension, and insulin resistance) that raises the risk for cardiovascular disease and type 2 diabetes mellitus (T2DM) ^1^. Globally, MetS represents an important and growing cardiometabolic concern in younger populations. A systematic review including more than 550,000 children and adolescents from 44 countries estimated the global prevalence of MetS among adolescents at 4.8% in 2020, corresponding to approximately 35.5 million affected adolescents worldwide ^2^. The burden appears particularly concerning in Saudi youth. A time-series analysis of 7,985 Saudi children and adolescents aged 12–18 years showed that MetS prevalence increased from 11.8% in 2010 to 20.1% in 2015 and 20.6% in 2019; the increase was especially marked among boys, rising from 7.8% to 25.3% over the same period ^3^. MetS has also been reported among Saudi school students, including female intermediate and secondary students^4^. The teenage years represent a period of important physiological change during which susceptibility to MetS and related cardiometabolic disorders may be established. Moreover, changes in socioeconomic conditions, urbanisation, and sedentary lifestyles have been suggested to increase MetS risk factor development among young people in Saudi Arabia ^5,6^. Traditional diets rich in fibre, fruits, and vegetables are gradually being substituted by Westernised dietary patterns with elevated intakes of saturated fats, sugars, and processed foods, which may contribute to increasing obesity rates and metabolic abnormalities among Saudi youth^7^.

The HGI is a biomarker representing interindividual variability in Hemoglobin glycation, not fully explained by average blood glucose levels ^8^. HGI was developed to quantify differences between an individual’s measured HbA1c and the HbA1c expected from the relationship between blood glucose and HbA1c in a reference population. It is calculated as the difference between observed HbA1c and HbA1c predicted from a regression equation based on a measure of glycemia, such as fasting plasma glucose (FPG). A positive HGI indicates that the observed HbA1c is higher than predicted for the corresponding glucose level, whereas a negative HGI indicates that it is lower than predicted. Thus, “glycation tendency” refers to an individual’s tendency to have relatively higher or lower HbA1c than expected from their glucose concentration. This variability may reflect biological factors affecting the glucose–HbA1c relationship, including erythrocyte lifespan and glucose transport into erythrocytes ^8,9^. In contrast to HbA1c, which reflects average blood glucose concentrations over the preceding two to three months, HGI characterises this interindividual variation in glycation and may therefore provide complementary information beyond HbA1c alone^9^. Higher HGI levels have been associated with adverse microvascular and macrovascular outcomes and all-cause mortality in some populations, independent of HbA1c ^10,11^. Correlations between HGI and inflammatory markers or oxidative stress have also been reported, suggesting a potential link between HGI and the pathophysiology of metabolic disorders ^12^. However, evidence regarding the association between HGI and MetS or its individual components remains inconsistent. Although several studies have reported positive associations, other studies have shown null or inverse associations, particularly among non-diabetic individuals and younger populations. The interplay between chronic inflammation, oxidative stress, and advanced glycation end-products (AGEs) in metabolic risk is complex and remains incompletely characterised ^13^.

Some studies suggest that HGI may be associated with components of MetS, with reports of higher HGI accompanying greater insulin resistance, dyslipidaemia, and adiposity in certain populations ^10,14^. Several biological pathways have been proposed to explain these associations, including alterations in erythrocyte metabolism, oxidative stress, and dysregulation of glucose transport mechanisms ^15^; however, these mechanisms were not directly assessed in the present study and remain hypothetical in this context. The involvement of HGI in endothelial dysfunction and arterial stiffness both relevant to cardiovascular complications of MetS has also been suggested ^16^. Furthermore, recent genome-wide evidence suggests that genetic variation contributes to interindividual differences in HGI, with potential heterogeneity by sex and ethnicity ^17^. Nevertheless, these proposed mechanisms have not been consistently demonstrated across all populations or age groups, and their relevance to adolescents remains uncertain.

Despite growing interest in HGI as a metabolic marker, data on this association are particularly limited among Saudi adolescents. Considering the high prevalence of obesity and metabolic diseases in this population, examining the cross-sectional association between HGI and MetS may contribute to metabolic risk characterisation in this group^3,5^. The influence of sex-specific hormonal changes during puberty on both HGI and MetS risk factors also warrants investigation ^18^. This study aimed to examine the cross-sectional association between HGI and MetS, and its individual components, among Saudi adolescents from Riyadh. Given the inconsistent evidence across populations and age groups, no directional association was prespecified. Sex-stratified analyses were conducted descriptively to examine potential sex-related differences in the observed associations between HGI and MetS-related variables; however, formal interaction testing was not performed.

## 2. Methodology

### 2.1. Participant Selection

In this cross-sectional study, a total of 2,342 adolescents (893 boys and 1,449 girls), with a mean age of 14.8 ± 1.7 years, were recruited between September 2019 and March 2021 from 60 preparatory and secondary schools across Riyadh, Saudi Arabia, in collaboration with the Ministry of Education. The study was approved by the Institutional Review Board of the College of Medicine, King Saud University (Approval No. E-21-6095; approved on January 18, 2019; amended on April 7, 2022). Written informed consent was obtained from parents or legal guardians, and assent was obtained from adolescents aged 12–17 years before participation. The study was conducted in accordance with the Declaration of Helsinki and its subsequent amendments.

A stratified random sampling approach was used. Schools were stratified according to geographic district and student sex, and schools were randomly selected within each stratum. Within each selected school, classes were randomly selected, and all eligible adolescents) aged 12–17 years(in the selected classes were invited to participate. Exclusion criteria included age <12 or >18 years, acute illness at the time of assessment, non-Saudi nationality, non-ambulatory status, and inability to provide informed consent. All consenting participants completed a structured questionnaire on demographic characteristics and medical history. Anthropometric measurements were obtained, and fasting blood samples were collected and stored at the Biobank of the Chair for Biomarkers of Chronic Diseases, King Saud University, Riyadh.

Recruitment commenced in September 2019, and the target sample size had been achieved before the implementation of major nationwide COVID-19-related school closures and movement restrictions in Saudi Arabia. Therefore, the potential influence of the COVID-19 pandemic on participant recruitment and the primary study sample was expected to be limited.

### 2.2. Anthropometry

All anthropometric measurements were obtained according to standardized procedures by certified nurses trained in epidemiological data collection and sample handling.

Body weight was measured to the nearest 0.1 kg using a Detecto balance beam scale (Detecto Scale Inc., Brooklyn), and height was measured in centimetres using a standardized stadiometer. Body mass index (BMI) was calculated as weight in kilograms divided by height in metres squared (kg/m²). Waist and hip circumferences were measured using a non-stretchable measuring tape, and the waist-to-hip ratio (WHR) was calculated.

Systolic blood pressure (SBP) and diastolic blood pressure (DBP) were measured using an automated oscillometric device. After participants had rested in a seated position for 10 minutes, three measurements were obtained, and the mean value was used for analysis.

### 2.3. Blood sample collection and biochemical analyses

Venous blood samples were collected in the morning after an overnight fast of 8–10 hours. Following collection, samples were centrifuged on-site to separate serum, placed on ice, and immediately transported to the CBCD laboratory at King Saud University, where they were stored at −80°C until analysis. Glycated hemoglobin (HbA1c) was measured using ion-exchange high-performance liquid chromatography (HPLC) on a D-10 Hemoglobin Analyzer (Bio-Rad Laboratories, Hercules). FPG, total cholesterol, high-density lipoprotein cholesterol (HDL-C), triglycerides (TG), serum calcium, phosphorus, iron, albumin, alkaline phosphatase (ALP), and alanine aminotransferase (ALT) were measured using commercially available biochemical assay kits: Glucose (catalogue no. 981379), Cholesterol (catalogue no. 981812), HDL-Cholesterol Plus (catalogue no. 981823), TG (catalogue no. 981301), Calcium (catalogue no. 981367), Phosphorus (catalogue no. 981890), Iron (catalogue no. 981236), Albumin (BCP; catalogue no. 981357), Alkaline Phosphatase (SCE; catalogue no. 981359), and ALT/GPT (catalogue no. 981769), respectively. All assays were performed on a Konelab 20XT clinical chemistry analyzer (Thermo Scientific, Vantaa, Finland) according to the manufacturer’s instructions. Bone-specific alkaline phosphatase (BAP) was measured using an electrochemiluminescence immunoassay on a Roche Elecsys Modular Analytics Cobas e411 automated analyzer (Roche Diagnostics, Mannheim, Germany). Serum parathyroid hormone (PTH) was measured using a Liaison 1–84 PTH immunoassay on a Liaison XL analyzer (DiaSorin, Italy). Serum total 25-hydroxyvitamin D concentrations were measured using a chemiluminescent immunoassay (DiaSorin, Saluggia, Italy), with inter-assay and intra-assay coefficients of variation of 10.6% and 5.4%, respectively, and a lower detection limit of 10 nmol/L.

### 2.4. Definition of MetS

MetS was defined according to established paediatric criteria ^19,20^. Participants were classified as having MetS if they met at least three of the following five criteria: abdominal obesity, defined as waist circumference ≥ 90th percentile for age and sex; elevated blood pressure, defined as systolic blood pressure and/or diastolic blood pressure ≥ 90th percentile for age, sex, and height; elevated FPG, defined as FPG ≥5.6 mmol/L or previously diagnosed diabetes; elevated TG, defined as TG ≥1.24 mmol/L; and low HDL-C, defined as HDL-C ≤1.03 mmol/L in boys and ≤ 1.29 mmol/L in girls. Participants were categorized into MetS and non-MetS groups for comparative analyses.

### 2.5. Calculation of HGI

HGI was calculated as the difference between observed and predicted HbA1c:^14^


HGI=Observed HbA1c−Predicted HbA1c

Predicted HbA1c was estimated using a linear regression equation based on FPG levels, derived from the current study population:


$$\begin{array}{c} Predicted\;HbA1c=3.573+(0.299\times FPG\left[mmol/L\right]) \end{array}$$

The regression model demonstrated a statistically significant positive association between FPG and HbA1c (*R²* = 0.522, p < 0.001). Participants were subsequently categorized into HGI tertiles to examine cross-sectional associations with MetS and its individual components.

### 2.6. Statistical analysis

All statistical analyses were performed using SPSS version 26.0 (IBM, Armonk). Continuous variables were assessed for normality and are presented as mean ± standard deviation (SD) for normally distributed data or median (interquartile range [IQR]) for non-normally distributed data. Categorical variables are presented as frequencies and percentages. Group comparisons for categorical variables were performed using the chi-square test or Fisher’s exact test, as appropriate. Normally distributed continuous variables were compared using the independent-samples *t* test, whereas non-normally distributed variables were compared using the Mann–Whitney U test. Binary logistic regression analyses were performed to examine the cross-sectional associations between HGI tertiles and MetS and its individual components. Unadjusted odds ratios (ORs) with 95% confidence intervals (CIs) were calculated, with the first HGI tertile used as the reference category. Pearson’s or Spearman’s correlation analyses, as appropriate, were performed to assess relationships between HGI, FPG, HbA1c, and metabolic parameters, stratified by sex. Statistical significance was set at a two-sided p-value <.05.

## 3. Results

This study examined the cross-sectional association between the HGI and MetS in 2,342 Saudi adolescents from Riyadh. Clinical characteristics are presented by sex and MetS status in Table 1. Associations between HGI tertiles and MetS components are presented in Table 2. Correlation coefficients between HGI, FPG, HbA1c, and other clinical and biochemical parameters, stratified by sex, are presented in Table 3.

**Table 1:** Clinical characteristics of subjects.

Parameters	All		Boys		Girls	
	Control	MetS	Control	MetS	Control	MetS
N	1329	1013	472	421	857	592
Age	14.7 ± 1.7	14.9 ± 1.7	14.6 ± 1.6	15.3 ± 1.5	14.8 ± 1.8	14.6 ± 1.7
BMI	21.9 ± 4.8	25.4 ± 6.1	21.5 ± 4.9	26.4 ± 5.9	22.3 ± 4.7	24.8 ± 6.2
BMI ZScore	−0.261 ± 0.84	0.345 ± 1.08	−0.357 ± 0.86	0.509 ± 1.04	−0.207 ± 0.83	0.227 ± 1.09
Waist	66.4 ± 13.9	77.5 ± 6.1	63.5 ± 20.2	78.5 ± 24.5	68.0 ± 8.2	76.7 ± 13.5
Hips	80.8 ± 17.2	91.7 ± 21.1	72.5 ± 22.4	86.9 ± 26.4	85.3 ± 11.1	95.1 ± 15.6
WHR	0.83 ± 0.09	0.85 ± 0.11	0.87 ± 0.07	0.90 ± 0.07	0.80 ± 0.09	0.81 ± 0.12
Systolic BP	110.0 ± 11.9	123.3 ± 17.3	113.9 ± 11.1	124.5 ± 14.0	107.9 ± 11.7	122.5 ± 19.3
Diastolic BP	68.9 ± 9.1	75.7 ± 12.5	66.1 ± 10.7	71.6 ± 106	70.5 ± 7.7	78.7 ± 12.9
Glucose0	4.98 ± 0.8	5.60 ± 1.7	5.1 ± 0.7	5.70 ± 1.6	4.92 ± 0.9	5.52 ± 1.7
HbA1C (%)	5.06 ± 0.7	5.23 ± 0.9	5.16 ± 0.7	5.37 ± 0.9	5.0 ± 0.7	5.14 ± 1.0
T-Cholersterol	4.68 ± 1.1	4.69 ± 1.1	4.36 ± 0.8	4.80 ± 1.2	4.86 ± 1.2	4.62 ± 0.9
HDL Cholesterol	1.20 ± 0.4	0.95 ± 0.3	1.10 ± 0.3	0.94 ± 0.3	1.26 ± 0.5	0.96 ± 0.3
Triglyceridies	0.96(0.8-1.23)	1.22(0.9–1.9)**	0.95(0.7–1.2)	1.05(0.7–2.1)**	0.97(0.8–1.3)	1.1(0.8–1.7)**
Ca	2.49 ± 0.3	2.51 ± 0.4	2.53 ± 0.4	2.58 ± 0.3	2.47 ± 0.3	2.47 ± 0.4
Pi	1.49 ± 0.4	1.45 ± 0.4	1.63 ± 0.4	1.53 ± 0.4	1.41 ± 0.3	1.40 ± 0.7
ALP (U/L)	59.7(41-86)	60.5(40-86)	65.5(44-87.9)	65.3(46-87)	57.7(39–85)	55.5(38-84)
VitD	32 (22-44.9)	30.3(22.6-39.3)**	38.9(30-49)	34.3(27-42.6)**	27.3(20–41.4)	26.9(20-36.5)
ALT_U_T	10.6(7.9-14.4)	14.1(10-19.1)**	12.3(10-16)	14.8(12-21)*	9.9(7.6–14.1)	11.9(10-16.9)**
Iron Ug/l	793(510-1100)	952(743-1210)**	1158(915-1435)	980(885-1264)	745(472–1036)	799(525-1032)
PTH (pg/ml)	23.6(16-32.2)	20.9(14.8-27.9)*	18.1(11-32.3)	18.8(13-26.9)	23.9(16–32)	23.5(16-29)
BAP (Ug/l)	13.4 ± 5.2	14.7 ± 4.5	18.1 ± 5.5	15.7 ± 4.2	13.1 ± 5.0	13.8 ± 4.5
ALB (BCG)	40.9 ± 3.3	43.3 ± 4.0	42.7 ± 3.6	44.9 ± 3.0	40.6 ± 3.1	40.1 ± 3.8
HGI index	−0.003 ± 0.52	−0.057 ± 0.6	0.076 ± 0.51	0.043 ± 0.63	−0.047 ± 0.51	−0.128 ± 0.55

Data are presented as n (%) for categorical variables and as mean ± SD or median (IQR) for continuous variables, as appropriate. Comparisons were made between the MetS and non-MetS groups within each stratum.

* p < 0.05.

** p < 0.01.

BMI, body mass index; WHR, waist-to-hip ratio; SBP, systolic blood pressure; DBP, diastolic blood pressure; FPG, fasting plasma glucose; HbA1c, glycated Hemoglobin; HDL-C, high-density lipoprotein cholesterol; TG, triglyceridesHemoglobin,; ALP, alkaline phosphatase; ALT, alanine aminotransferaseHemoglobin glycation index; PTH, parathyroid hormone; BAP, bone alkaline phosphatase; ALB, albumin; HGI, Hemoglobin glycation index; MetS, metabolic syndrome.

**Table 2:** Logistic regression analysis dependent metabolic components and MetS, HGI 1^st^ tritile reference.

Parameters	N	Unadjusted Odd Ratio (95% CI)	*P*-Value	Adjusted Odd Ratio (95% CI)	*P*-Value
Abdominal obesity					
HGI T1	125 (15.5)	1		1	
HGI T2	122 (15.8)	1.02 (0.78-1.34)	.87	0.89 (0.64-1.24)	.49
HGI T3	153 (19.9)	1.35 (1.04-1.75)	.02	0.86 (0.62-1.18)	.34
Hypertension					
HGI T1	193 (24.0)	1		1	
HGI T2	157 (20.4)	0.81 (0.64-1.03)	.08	0.76 (0.59-0.97)	.03
HGI T3	179 (23.3)	0.96 (0.76-1.21)	.74	0.79 (0.62-1.01)	.061
High glucose					
HGI T1	222 (27.6)	1		1	
HGI T2	91 (11.8)	0.35 (0.27-0.46)	<.001	0.33 (0.25-0.43)	<.001
HGI T3	133 (17.3)	0.55 (0.43-0.70)	<.001	0.47 (0.36-0.61)	<.001
High Trigly					
HGI T1	133 (16.5)	1		**1**	
HGI T2	124 (16.1)	0.97 (0.74-1.27)	.81	0.90 (0.68-1.19)	.453
HGI T3	181 (23.6)	1.56 (1.21-1.99)	<.001	1.29 (0.99-1.68)	.057
Low HDL					
HGI T1	590 (73.4)	1		1	
HGI T2	557 (72.3)	0.94 (0.76-1.18)	.64	1.06 (0.84-1.33)	.63
HGI T3	477 (62.1)	0.60 (0.48-0.74)	<.001	0.70 (0.56-0.87)	.002
MetS					
HGI T1	387 (48.1)	1		1	
HGI T2	299 (38.8)	0.68 (0.56-0.84)	<.001	0.62 (0.49-0.76)	<.001
HGI T3	327 (42.6)	0.79 (0.65-0.98)	.027	0.61 (0.49-0.75)	<.001

Data are presented as odds ratios with 95% confidence intervals. The first HGI tertile was used as the reference category. Abdominal obesity, hypertension, high fasting glucose, high triglycerides, and low HDL-C represent the five individual MetS components; MetS was defined as meeting ≥3 criteria.

P-value significant at .05.

**Table 3:** Correlation coefficients of hemoglobin glycation index (HGI), fasting glucose, and HbA1c with clinical and biochemical parameters in adolescents, stratified by sex.

Parameters	ALL			Boys			Girls		
	HGI index	Glucose	HbA1C (%)	HGI index	Glucose	HbA1C (%)	HGI index	Glucose	HbA1C (%)
Age	0.02	0.00	0.01	0.02	0.04	0.02	0.00	−0.02	−0.01
BMI	0.096**	0.053*	0.091**	0.097**	0.05	0.097**	0.088**	0.052*	0.081**
BMI ZScore	0.096**	0.053*	0.091**	0.097**	0.05	0.097**	0.088**	0.052*	0.081**
Waist	0.00	0.043*	0.02	−0.03	0.05	−0.01	0.05	0.053*	0.072**
Hips	−0.048*	0.02	−0.03	−0.03	0.05	−0.01	−0.01	0.04	0.01
WHR	0.081**	0.049*	0.092**	−0.03	0.01	0.00	0.073**	0.02	0.075**
Systolic BP	−0.02	0.059**	0.02	0.04	0.068*	0.067*	−0.080**	0.04	−0.03
Diastolic BP	−0.042*	0.04	−0.02	0.00	0.108**	0.070*	−0.02	0.03	−0.03
T-Cholersterol	0.055**	0.093**	0.085**	−0.079*	0.201**	0.06	0.157**	0.05	0.115**
HDL Cholesterol	0.063**	0.01	0.044*	−0.06	0.073*	0.00	0.139**	0.00	0.083**
Triglyceridies	0.066**	0.185**	0.165**	−0.04	0.251**	0.138**	0.127**	0.129**	0.166**
Ca	−0.046*	0.03	−0.02	−0.066*	0.06	−0.01	−0.059*	0.00	−0.053*
Pi	0.063**	−0.052*	0.02	0.00	−0.109**	−0.05	0.066*	−0.052*	0.03
ALP (U/L)	0.067**	0.075**	0.087**	−0.05	0.126**	0.04	0.115**	0.04	0.099**
VitD	0.099**	−0.01	0.04	0.01	−0.104**	−0.06	0.110**	0.01	0.05
ALT_U_T	−0.01	0.116*	0.131*	−0.01	0.14	0.15	0.00	0.08	0.11
Iron Ug/l	−0.112*	0.02	−0.06	0.02	−0.13	−0.06	−0.10	0.01	−0.05
PTH (pg/ml)	0.142**	−0.05	0.07	0.17	−0.03	0.15	0.10	−0.03	0.04
BAP (Ug/l)	0.09	0.110*	0.133*	0.09	0.04	0.11	0.128*	0.11	0.146*
ALB (BCG)	−0.272**	0.00	−0.195**	−0.389**	0.09	−0.275**	−0.11	−0.145*	−0.182**
HGI index	1.00			1.00			1.00		
Glucose0	−0.042*	1.00		−0.142**	1.00		0.00	1.00	
HbA1C (%)	0.780**	0.522**	1.00	0.786**	0.478**	1.00	0.771**	0.539**	1.00

Pearson’s correlation coefficients are reported for normally distributed variables and Spearman’s rho for non-normally distributed variables.

p < 0.05; p < 0.01.

Given the large study sample (n = 2,342), statistically significant correlations should be interpreted in relation to their magnitude; most were weak ( *r* < .15). No formal HGI × sex interaction analysis was performed; sex-stratified findings are exploratory.

* and ** denote significance at 0.05 and 0.01, respectively.

Clinical characteristics of study participants by sex and MetS status are shown in Table 1. Of the 2,342 adolescents, MetS was identified in 1,013 (43.3%), with a higher prevalence in boys (47.1%) than in girls (40.9%). Participants with MetS had significantly higher BMI, BMI Z-scores, waist circumference, hip circumference, and WHR than those without MetS (p < 0.01). Both systolic and diastolic blood pressure were significantly higher in the MetS group, with more pronounced elevations in boys than in girls. FPG and HbA1c levels were significantly higher in the MetS group, with boys showing a greater elevation in FPG than girls. Dyslipidaemia was evident in the MetS group, with significantly higher TG and lower HDL-C (p < 0.01). Serum vitamin D levels were significantly lower, whereas ALT levels were significantly higher, in participants with MetS (p < 0.01). HGI was significantly lower in adolescents with MetS than in the non-MetS group (−0.057 ± 0.60 vs −0.003 ± 0.52, p < 0.05), with the largest difference observed among girls (−0.128 ± 0.55 vs −0.047 ± 0.51, p < 0.05).

Figure 1 presents mean ± SD values for FPG and HbA1c across HGI tertiles (T1, T2, and T3) in participants with MetS and those without MetS. Trend analyses indicated statistically significant increases in FPG and HbA1c across HGI tertiles in both groups (*P*-trend < .001). Participants with MetS had higher mean FPG and HbA1c values than those without MetS across tertiles.

**Figure 1.**
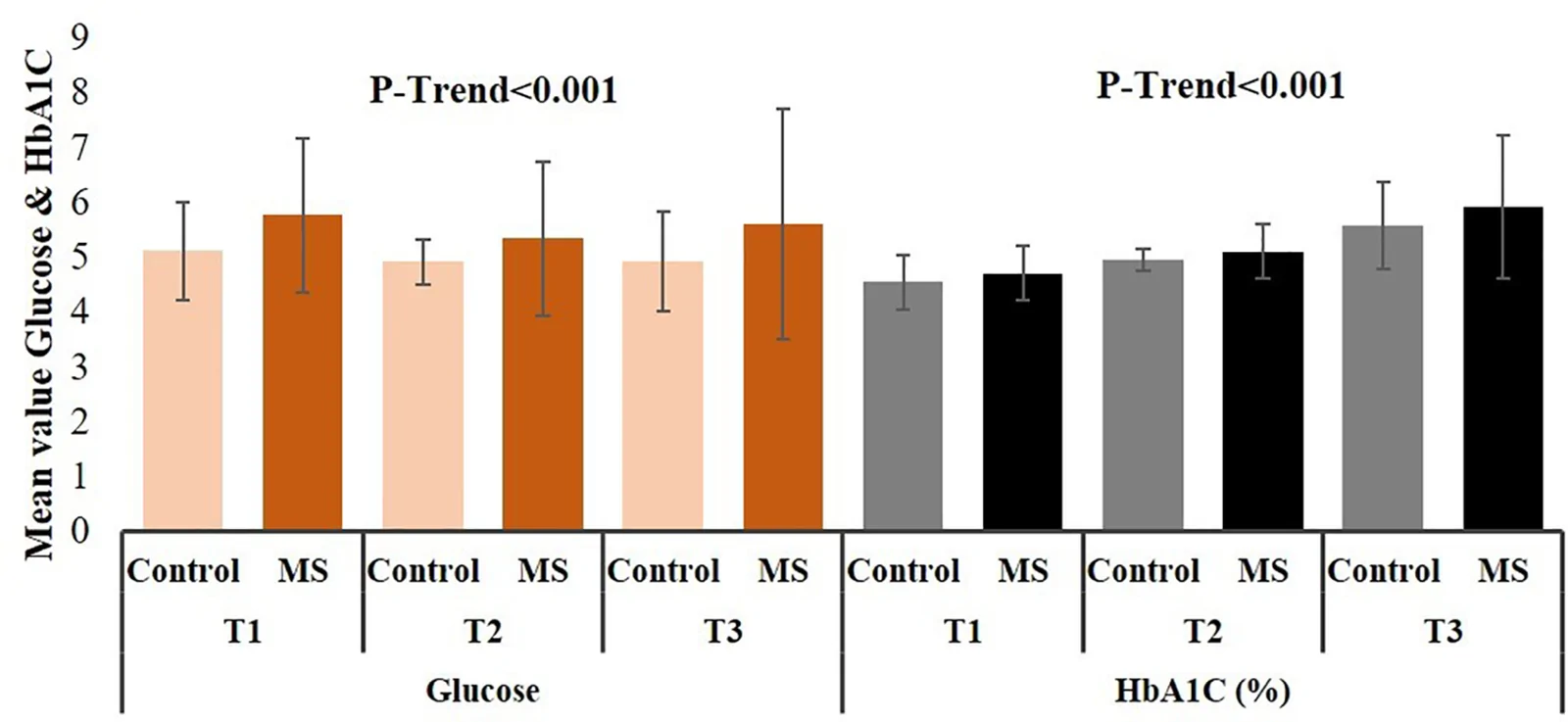
Mean ± SD fasting plasma glucose (FPG) and glycated Hemoglobin (HbA1c) values across hemoglobin glycation index (HGI) tertiles in participants with and without metabolic syndrome (MetS). *P* for trend were < .001 for FPG and HbA1c across HGI tertiles within both MetS and non-MetS groups.

Table 2 presents the unadjusted logistic regression analysis of associations between HGI tertiles and MetS and its individual components, with the first tertile (T1) as the reference category. In the overall sample, HGI T3 was associated with higher odds of abdominal obesity (OR = 1.35, 95% CI: 1.04–1.75, p = 0.023) and high TG (OR = 1.56, 95% CI: 1.21–1.99, p < 0.001), and with lower odds of low HDL-C (OR = 0.60, 95% CI: 0.48–0.74, p < 0.001). Both HGI T2 and T3 were associated with lower odds of high FPG (T2: OR = 0.35, 95% CI: 0.27–0.46; T3: OR = 0.55, 95% CI: 0.43–0.70; both p < 0.001). Compared with T1, HGI T2 and T3 were also associated with lower odds of MetS (T2: OR = 0.68, 95% CI: 0.56–0.84, p < 0.001; T3: OR = 0.79, 95% CI: 0.65–0.98, p = 0.027).

In sex-stratified analyses, associations differed between boys and girls. Among boys, HGI T2 was associated with lower odds of abdominal obesity, high FPG, high TG, and MetS, whereas HGI T3 was associated with lower odds of high FPG and low HDL-C. Among girls, HGI T2 and T3 were associated with lower odds of high FPG, whereas both tertiles were associated with higher odds of high TG. HGI T3 was associated with lower odds of low HDL-C and MetS among girls. These sex-stratified findings should be interpreted descriptively because formal interaction testing was not performed.

Pearson and Spearman correlation coefficients between HGI, FPG, HbA1c, and clinical and biochemical parameters are presented in Table 3, stratified by sex. Given the large study sample (n = 2,342), several small correlation coefficients reached statistical significance; however, most statistically significant correlations were weak in magnitude (|*r*| < 0.15). In the overall sample, HGI showed weak positive correlations with BMI (r = 0.096, p < 0.01), BMI Z-score (r = 0.096, p < 0.01), WHR (r = 0.081, p < 0.01), total cholesterol (r = 0.055, p < 0.01), HDL-C (r = 0.063, p < 0.01), TG (r = 0.066, p < 0.01), alkaline phosphatase (r = 0.067, p < 0.01), vitamin D (r = 0.099, p < 0.01), and parathyroid hormone (r = 0.142, p < 0.01). Significant negative correlations were observed with serum albumin (*r* = −0.272, p < 0.01) and serum iron (*r* = −0.112, p < 0.05). HbA1c showed a strong positive correlation with HGI across all groups (r = 0.780, p < 0.01), which is expected because HGI is derived partly from observed HbA1c. Sex-stratified analyses showed differing correlation patterns between boys and girls; however, no formal HGI × sex interaction analysis was conducted, and these findings should be interpreted as exploratory.

Figure 2 illustrates the linear relationship between FPG and HbA1c in the study population. HbA1c increased significantly with increasing FPG levels (*R*² = 0.522, p < 0.001). The fitted regression equation, Predicted HbA1c = 3.573 + (0.299 × FPG [mmol/L]), was used to estimate predicted HbA1c values for calculation of HGI.

**Figure 2.**
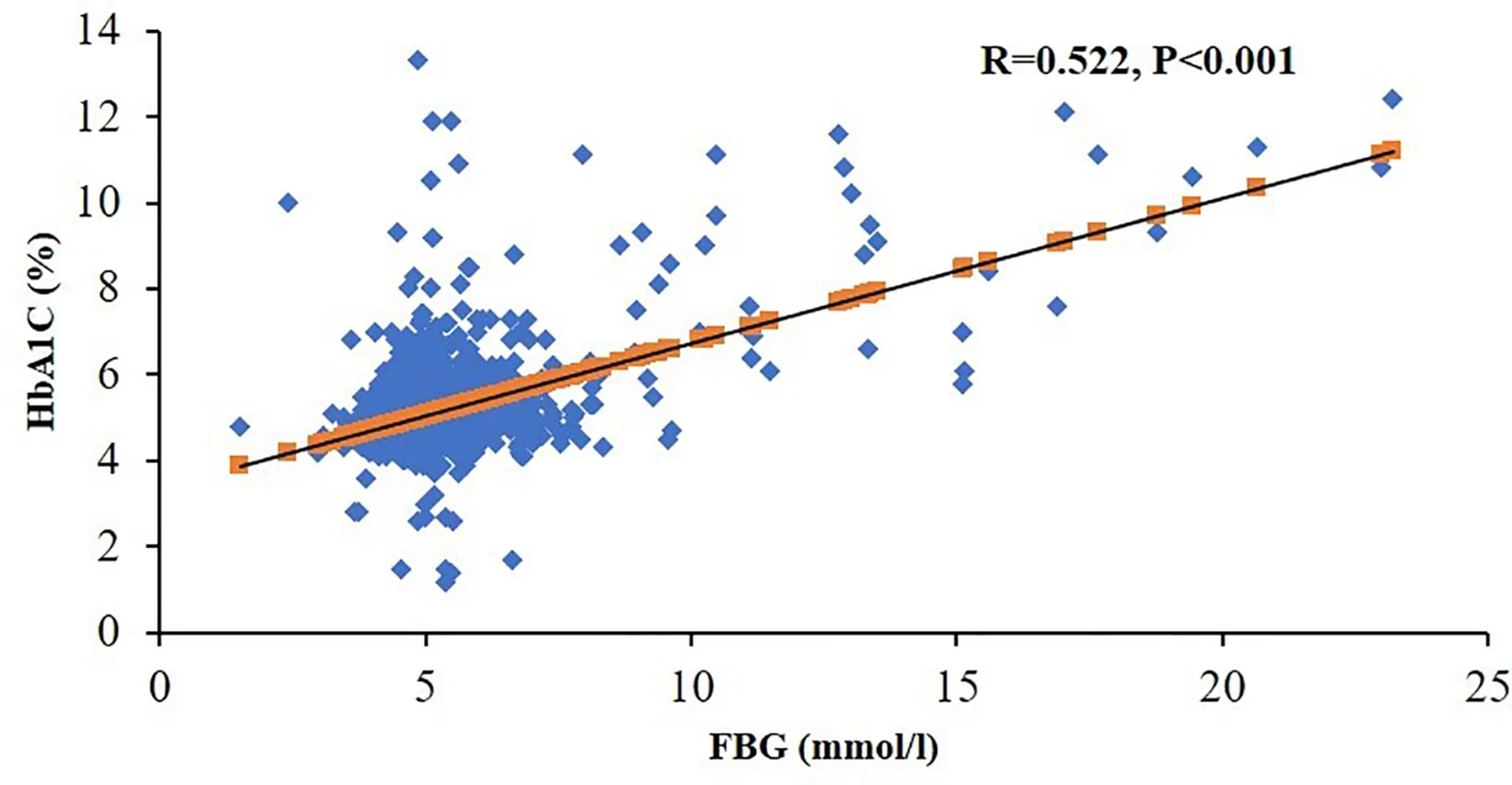
Linear regression of glycated Hemoglobin (HbA1c) on fasting plasma glucose (FPG) in the study population (The fitted regression equation was: Predicted HbA1c = 3.573 + [0.299 × FPG (mmol/L)]; R² = 0.522, p < 0.001).

## 4. Discussion

This study investigated the cross-sectional association between HGI and MetS in a large cohort of 2,342 Saudi adolescents from Riyadh, with analyses stratified by sex. The primary finding was an inverse association between HGI and MetS in the unadjusted analyses, which did not support the original hypothesis of a positive association. However, because the logistic regression analyses were unadjusted, these findings should not be interpreted as evidence of an independent association. This unexpected finding highlights the complexity of the HGI–MetS relationship in adolescents and is contextualised below in relation to prior literature, biological considerations, and study limitations.

The prevalence of MetS among adolescents in this study was 43.3% (1,013 out of 2,342), with boys showing a higher prevalence (47.1%) than girls (40.9%). This prevalence is notably higher than most global estimates for adolescent MetS and likely reflects the specific sociodemographic and lifestyle characteristics of school-attending adolescents in Riyadh ^3,4,21^, although pandemic-related increases in obesity and MetS among children and adolescents have also been reported elsewhere ^22^, including high rates of sedentary behaviour, dietary change, and obesity; these findings should not be extrapolated beyond this recruitment context^5,7^. The observed sex difference is consistent with earlier studies reporting more adverse metabolic profiles in adolescent boys, attributed in part to differences in fat distribution and insulin sensitivity ^23,24^. Prior literature has proposed that higher lipolytic activity driven by testosterone and lower adiponectin levels may contribute to greater visceral fat deposition in boys, while oestrogen may have a partially protective metabolic role in females through effects on insulin sensitivity and lipid metabolism, though these hormonal variables were not assessed in the current study ^25-27^.

Adolescents with MetS had significantly higher BMI, BMI Z-scores, waist circumference, hip circumference, and WHR compared with those without MetS, consistent with the central role of adiposity in MetS ^28,29^. In Saudi children with obesity, a lifestyle education programme was associated with improvements in several cardiometabolic indicators, further supporting the relevance of obesity-related lifestyle factors to cardiometabolic risk in this population ^29^. Visceral adiposity has been linked in prior literature to elevated inflammatory cytokines and adipokine dysregulation promoting insulin resistance and systemic inflammation; however, these inflammatory markers were not measured in the current study ^30,31^. The association between adiposity, hepatic insulin resistance, and dyslipidaemia has been well described in literature ^32^.

Significantly higher systolic and diastolic blood pressure were observed in individuals with MetS, consistent with prior findings on the association between excess adiposity and blood pressure elevation through multiple pathways including sympathetic nervous system activation and altered sodium retention ^14^. Adipose tissue-derived vasoactive factors have been implicated in obesity-related hypertension in prior literature, though these were not measured here ^33^. Boys showed a more pronounced blood pressure increase than girls, which is consistent with prior reports, although the underlying mechanisms were not directly examined in this study^34^.

Glycaemic parameters (FPG and HbA1c) were significantly higher in adolescents with MetS than in those without MetS, and boys showed a more pronounced elevation in FPG. Prior literature has proposed that puberty-associated insulin resistance may be more pronounced in boys than girls, which may contribute to this pattern; however, insulin resistance was not directly measured in the current study (e.g., HOMA-IR was not available)^35^.

Dyslipidaemia was a prominent feature of MetS in this sample, with significantly higher TG and lower HDL-C; boys showed a greater degree of lipid perturbation than girls, consistent with prior literature ^36^. Regarding the primary outcome, HGI was significantly lower in individuals with MetS than without MetS (−0.057 ± 0.60 vs −0.003 ± 0.52, *p* < 0.05), with the greatest difference in girls (−0.128 ± 0.55 vs −0.047 ± 0.51, *p* < 0.05). Consistent with these group-level findings, higher HGI tertiles were associated with lower odds of MetS and high FPG in the overall unadjusted analyses; however, the sex-stratified associations were not uniform. This inverse association contrasts with the majority of prior HGI studies, which have generally reported positive associations between HGI and adverse metabolic outcomes in adult populations ^37^. The following section discusses plausible but unconfirmed explanations for this discrepancy.

Several hypothetical mechanisms may potentially explain the observed inverse association, but it is important to emphasise that none of the following biological variables were directly measured in this study; these explanations are speculative and are offered as hypotheses for future investigation. First, oxidative stress and chronic inflammation in MetS have been proposed in prior literature to accelerate erythrocyte turnover, potentially lowering HbA1c despite elevated glucose concentrations ^20,38^. Second, insulin-resistant states may alter erythrocyte membrane properties, potentially affecting glycation efficiency ^39^. Third, greater glucose variability in insulin-resistant adolescents may contribute to discordance between HbA1c and mean glucose levels ^40^. Fourth, sex-based differences in glycation efficiency, possibly related to oestrogenic effects on glucose uptake and erythrocyte turnover, may partly account for the more pronounced HGI difference in girls, though this remains entirely speculative without hormonal measurements ^41,42^. The discrepancy with prior literature may also reflect age-specific differences in erythrocyte physiology, the predominantly normoglycaemic profile of this adolescent cohort (in whom MetS is largely driven by obesity and lipid components rather than hyperglycaemia), ethnic or population-specific differences, or methodological factors, including the population-specific HGI regression equation.

The current findings indicate an inverse cross-sectional association between HGI and MetS in Saudi adolescents from Riyadh, which differs from patterns reported in most prior HGI studies conducted predominantly in adult populations. This observation should be interpreted as descriptive and hypothesis-generating rather than causal or independent. This discrepancy highlights the importance of examining HGI–MetS associations separately in adolescent and paediatric populations. The biological significance of HGI may differ across age groups and metabolic contexts.

The strengths of this study include its large study sample (*n* = 2,342), which enhances statistical power; the use of stratified random sampling across 60 schools in Riyadh; standardised biochemical measurements; and sex-stratified analyses. Nevertheless, the study has several important limitations. First, the cross-sectional design precludes causal or temporal inference. Second, dietary intake and physical activity were not directly assessed. Third, the study was restricted to secondary school adolescents in Riyadh; findings should not be generalized to adolescents from other regions of Saudi Arabia, other Arab countries, or other ethnic groups. Fourth, insulin resistance was not directly measured (e.g., HOMA-IR was not available). Fifth, recruitment commenced before the COVID-19 pandemic, and the target sample size had been achieved before major COVID-19-related restrictions; therefore, the influence of the pandemic on recruitment and the primary study sample was expected to be limited. Nevertheless, accelerated increases in BMI among children and adolescents during the COVID-19 pandemic have been reported in longitudinal data, and pandemic-related changes in lifestyle behaviours may have influenced cardiometabolic risk profiles in some participants.^22^. Sixth, pubertal status was not systematically assessed, which is a significant limitation given that puberty profoundly influences both metabolic parameters and Hemoglobin glycation. Seventh, medication use (e.g., antihypertensives, lipid-lowering agents, oral contraceptives) was not reported; potential effects on metabolic parameters could not be accounted for. Eighth, the handling of missing data was not documented in the available study records. Ninth, residual confounding from unmeasured dietary, lifestyle, genetic, and socioeconomic factors is also possible. Tenth, no formal HGI × sex interaction analysis was conducted; sex-stratified findings are therefore exploratory. Eleventh, the regression equation used to estimate predicted HbA1c was derived from the current study population; therefore, HGI estimates should be interpreted as population-specific. Twelfth, no formal correction for multiple comparisons was applied, and statistically significant findings should be interpreted cautiously.

### Future research

Future longitudinal studies are needed to clarify the directionality of the HGI–MetS association in adolescents. These studies should incorporate direct measures of insulin resistance, inflammatory and oxidative stress markers, erythrocyte turnover, and pubertal status. Validation in broader adolescent populations and formal assessment of HGI × sex interactions are also warranted.

## Conclusion

This cross-sectional study of 2,342 Saudi adolescents from Riyadh found that higher HGI tertiles were associated with lower odds of MetS in unadjusted analyses, contrary to the original hypothesis of a positive association. Because these associations were unadjusted, they should not be interpreted as independent associations. The findings indicate that the cross-sectional relationship between HGI and MetS in adolescents is complex and may differ from patterns reported in predominantly adult populations. Sex-stratified differences in the associations between HGI and metabolic parameters were observed; however, these should be interpreted cautiously because formal HGI × sex interaction analyses were not conducted.

The study also confirmed the expected cross-sectional associations of obesity, elevated blood pressure, dyslipidaemia, and elevated FPG with MetS. Given the cross-sectional design, causal inferences cannot be drawn, and the findings apply specifically to school-attending adolescents in Riyadh, Saudi Arabia.

## Author Contributions

**Writing – original draft:** Sobhy M. Yakout

**Writing – review & editing:** Sobhy M. Yakout

**Formal analysis:** Malak N.K. Khattak

**Conceptualization:** Abdullah M Alnaami, Nasser M. Al-Daghri
